# Thoracic fluid content: a novel parameter for predicting failed weaning from mechanical ventilation

**DOI:** 10.1186/s40560-020-00439-2

**Published:** 2020-03-05

**Authors:** Shymaa Fathy, Ahmed M. Hasanin, Mohamed Raafat, Maha M. A. Mostafa, Ahmed M. Fetouh, Mohamed Elsayed, Esraa M. Badr, Hanan M. Kamal, Ahmed Z. Fouad

**Affiliations:** Department of Anesthesia and Critical Care Medicine, Faculty of Medicine, Cairo University, 01 Elsarayah Street, Elmanyal, Cairo, 11559 Egypt

**Keywords:** Thoracic fluid content, Electrical cardiometry, Weaning, Mechanical ventilation, Impaired cardiac contractility

## Abstract

**Background:**

Weaning of patients from the mechanical ventilation remains one of the critical decisions in intensive care unit. This study aimed to evaluate the accuracy of thoracic fluid content (TFC) as a predictor of weaning outcome.

**Methods:**

An observational cohort study included 64 critically ill surgical patients who were eligible for extubation. Before initiating the spontaneous breathing trial, the TFC was measured using the electrical cardiometry technology. Patients were followed up after extubation and divided into successful weaning group and failed weaning group. Both groups were compared according to respiratory and cardiovascular parameters. Receiver operating characteristic (ROC) curves were constructed to evaluate the ability of TFC to predict weaning outcome.

**Results:**

The number of successfully weaned patients was 41/64 (64%). Twenty (31%) patients had impaired cardiac contractility, and of them, 13/20 (64%) patients were successfully extubated. Both groups, successful weaning group and failed weaning group, were comparable in most of baseline characteristics; however, the TFC was significantly higher in the failed weaning group compared to the successful weaning group. The area under the ROC curves (AUCs) showed moderate predictive ability for the TFC in predicting weaning failure (AUC [95% confidence interval] 0.69 [0.57–0.8], cutoff value > 50 kΩ^−1^), while the predictive ability of TFC was excellent in the subgroup of patients with ejection fraction < 40% (AUC [95% confidence interval 0.93 [0.72–1], cutoff value > 50 kΩ^−1^).

**Conclusions:**

Thoracic fluid content showed moderate ability for predicting weaning outcome in surgical critically ill patients. However, in the subgroup of patients with ejection fraction less than 40%, TFC above 50 kΩ^−1^ has an excellent ability to predict weaning failure.

## Introduction

Weaning of patients from the mechanical ventilation remains one of the critical decisions in intensive care unit. Earlier patient weaning from mechanical ventilation is recommended to avoid complications of prolonged mechanical ventilation; however, premature weaning might result in extubation failure which is, independently, associated with poor outcomes [1]. Screening for eligibility is the first step in weaning process, followed by the spontaneous breathing trial (SBT) [2]. Various indices should be checked carefully before starting SBT to ensure adequate oxygenation, ventilation, and airway reflexes [2]. However, nearly one-third of patients fail and are reintubated despite fulfillment of all the current weaning pre-requisites [3, 4]. The absence of the ideal weaning parameter till now might be due to the heterogeneity of critically ill patients which impairs the predictive accuracy of the available indices in different patient subgroups.

Nowadays, there is an increasing interest in cardiac factors, such as lung congestion and hypervolemia, as contributing elements in weaning failure [5, 6]. Various measures had been previously reported for evaluation of volume status before the SBT aiming to identify patients who would benefit from diuretic therapy [5]. Most of the previously used measures required either frequent blood sampling, such as brain natriuretic peptide [7], or expert operator, such as echocardiography [8]. The electrical cardiometry-derived thoracic fluid content (TFC) is measured through the impedance cardiography technology; TFC is assessed through the changes in the impedance of thoracic tissue to the electric current [9]. Thoracic fluid content represents the whole (extravascular, intravascular, and intrapleural) fluid component in the thorax; thus, TFC was postulated to provide an estimation of the extravascular lung water. Our group had recently reported that TFC could detect pulmonary edema in parturients with pre-eclampsia [10]. We had also found that TFC correlated perfectly with extravascular lung water in pre-eclamptic patients [10]. In this study, we aimed to evaluate the accuracy of TFC, as an estimate of lung congestion, in predicting weaning outcome in critically ill patients.

## Materials and methods

We conducted this prospective observational study in surgical intensive care unit (ICU), over a 6-month period, after being approved by the Cairo University Research Ethics Committee. Informed consent was obtained from patients’ next-of-kin before inclusion in the study. All patients who were mechanically ventilated for more than 48 h were consecutively included in the study. Recruitment of the patients was continued until reaching the minimum required number of patients with successful weaning and failed weaning.

Patients younger than 18 years and patients with pneumothorax, pleural, or pericardial effusion were excluded from the study. Patients with injuries, burns, or wounds which precluded the proper application of the device electrodes were also excluded from the study.

### Weaning readiness assessment

The decision of (readiness-to-wean) was performed by the attending ICU physician according to the ICU protocol [2, 11]. Criteria of weaning included resolution of the primary cause of intubation, adequate cough without excessive tracheobronchial secretions, PaO_2_ > 60 mmHg with positive end-expiratory pressure ≤ 8 cmH_2_O, fraction of inspired oxygen ≤ 0.4, respiratory rate < 30 per minute, appropriate *p*H for patients’ baseline respiratory status, and stable cardiovascular status.

### Spontaneous breathing trial

For performing the SBT, the ventilator was set to the pressure support mode, with a pressure support of 5 cmH_2_O and positive end-expiratory pressure of 5 cmH_2_O. The SBT continued for 30 min, and then the weaning parameters were checked again. The decision of extubation was taken by the ICU physician who was blinded to the TFC measurements.

Weaning failure was defined as reintubation within 48 h after extubation due the presence of one or more of the following criteria: tachypnea (respiratory rate more than 35 breaths per minute), oxygen saturation less than 90% or PaO_2_ less than 60 mmHg on a fraction of inspired oxygen of 40%, apparent increase in accessory respiratory muscle activity, and evident facial signs of respiratory distress [1].

Thoracic fluid content was measured using electrical cardiometry device (ICON^R^ monitor: Osypka Medical, Inc., La Jolla, California and Berlin). The ICON device was connected to four electrocardiogram electrodes which were placed over patients’ skin after cleaning with alcohol at the neck below the left ear, just above the left clavicular midpoint, and two electrodes at left mid-axillary line one at the level of the xiphoid process, and the other electrode 5 cm below this point. The TFC was observed for 30 s and the average of the highest and lowest values was recorded.

Trans-thoracic echocardiogram was performed before the initiation of the SBT by a trained attending ICU physician. Ejection fraction was estimated by eyeballing, and patients were divided into cardiac patients (whom ejection fraction is < 40%) and non-cardiac patients (whom ejection fraction is ≥ 40%).

Patients were divided into successful weaning group and failed weaning group. Both groups were compared according to the study outcomes. Further subgroup analysis was performed within cardiac and non-cardiac patients.

### Primary outcome

The area under receiver operating characteristic curve (AUC) for TFC to predict weaning failure. TFC was recorded 5 min before initiation of SBT.

### Secondary outcomes

Hemodynamic data: heart rate, systolic blood pressure, and central venous pressure. Data were recorded 5 min before initiation of the SBT.

#### Lung compliance and minute ventilation

Rapid shallow breathing index (RSBI): calculated by dividing respiratory rate/tidal volume.

Other data including severity scores, pH, PCO_2_, serum HCO_3_, serum lactate, hemoglobin level, fluid balance 24 h before the onset of the SBT, days of mechanical ventilation, and number of previous SBT. All data were obtained 5 min before initiation of the SBT.

### Statistical analysis and sample size calculation

The sample size was calculated using MedCalc version 12.1.4.0 (MedCalc Software bvba, Mariakerke, Belgium) to detect AUC of 0.75 for TFC to predict weaning failure and the null hypothesis of AUC was set at 0.5. The study power was set at 80% and the alpha error was set at 0.05. The minimum number of patients was calculated to be 38, with at least 19 patients with failed weaning and 19 patients with successful weaning.

Data were presented as the means (standard deviations), medians (quartiles), and frequencies (%) as appropriate. Data were checked for normality using the Shapiro-Wilk test. Unpaired Student’s *t* test or Mann-Whitney *U* test were used to analyze continuous variables, while the categorical variables were analyzed using either chi-squared test or Fisher’s exact test as appropriate. MedCalc version 12.1.4.0 (MedCalc Software bvba, Mariakerke, Belgium) generated receiver operating characteristic curves and the AUC were calculated. Values with the highest sensitivity and specificity (Youden index) were obtained. The risk factors for failed weaning which showed *p* values less than 0.2 were enrolled in a multivariate analysis model to determine the independent risk factors for failed weaning. The level of significance was set at *p* ≤ 0.05.

## Results

We screened 71 consecutive patients. Three patients were excluded because they met any of the exclusion criteria (pleural effusion–pneumothorax–chest lesions which impaired cardiometry signals), and 68 patients were included in the study. Four patients were not extubated due to failed SBT and 64 patients completed the SBT and were available for the final analysis. Forty-one (64%) patients were successfully extubated; whereas, 23 (36%) patients had failed all of them were re-intubated within 48 h. Twenty (31%) patients showed reduced cardiac contractility. Among the subgroup of patients with impaired cardiac contractility, 13 (65%) patients were successfully extubated while 7 (35%) patients failed.

Both groups of patients, failed weaning group and successful weaning group, were comparable in demographic data, duration of ventilation before SBT, number of previous weaning trials, pre-SBT balance, arterial blood gas values, lung mechanics, and hemodynamic variables. However, the TFC was higher in the failed SBT group compared to the successful SBT group (61 ± 26 kΩ^−1^ versus 45 ± 14 kΩ^−1^, *p* = 0.002) (Tables 1, 2, and 3). Multivariate analysis revealed that the following factors were independently associated with failed weaning: higher age, higher TFC, and higher serum HCO3 with odds ratio (95% confidence interval [CI]) of 1.06 (1.02–1.11), 1.04 (1.01–1.08), 1.17 (1.01–1.38).

**Table 1:** Demographic data and baseline characteristics. Data are presented as mean (standard deviation), median (quartiles), and frequency (%)

	Failed weaning (*n* = 23)	Successful weaning (*n* = 41)	*p* value
Age (years)	54.4 (20.2)	43.9 (15.6)	0.057
Male gender (%)	19 (83%)	21 (51%)	0.012*
APACHE II	18 (12, 23)	19 (15, 22)	0.17
Duration of mechanical ventilation before SBT (days)	3 (2, 6)	3 (2, 4)	0.17
Number of previous SBT	1 (1, 2)	1 (0, 1)	0.32
Pre-SBT day balance (mL)	215 (1102)	20 (929)	0.899
SBT day balance (mL)	− 262 (713)	− 234 (782)	0.481
Blood hemoglobin (g dL^−1^)	10(1.5)	10.6(2.2)	0.046*
Chronic obstructive pulmonary disease (%)	5 (22%)	1 (2%)	0.02*
Abdominal surgery (%)	10 (44%)	20 (49%)	0.44

*APACHE* Acute physiology and chronic health evaluation, *SBT* spontaneous breathing trial

*Denotes statistical significance

**Table 2:** Arterial blood gases and respiratory mechanics. Data are means (standard deviation) or medians (quartiles)

	Failed weaning (*n* = 23)	Successful weaning (*n* = 41)	*p* value
P/F ratio	296.7 (75.4)	338 (87.4)	0.09
PaCO_2_ (mmHg)	34 (28,36)	31 (28.5, 35.5)	0.18
*p*H	7.47 (0.07)	7.45 (0.07)	0.66
HCO_3_ (mEq L^−1^)	26.4 (5.7)	23.7(4.6)	0.14
Lactate (mmol L^−1^)	1.3 (1, 1.8)	1.3 (0.9, 1.65)	0.69
RSBI	40 (29, 60)	40 (29.3, 56)	0.87
Minute ventilation (L min^−1^)	9.8 (2.7)	9.1 (2.4)	0.22
Compliance (dynes)	67 (45, 91)	62.8 (44.5, 60)	0.69
TFC (kΩ^−1^)	61 (26)	45 (14)	0.002*

*P/F ratio* PaO_2_/fraction of inspired oxygen, *TFC* thoracic fluid content, *RSBI* rapid shallow breathing index

*Denotes statistical significance

**Table 3:** Hemodynamic variables before SBT. Data are medians (quartiles) and means (standard deviations)

	Failed weaning (*n* = 23)	Successful weaning (*n* = 41)	*p* value
TFC (kΩ^−1^)			
Non-cardiac patients	47 (34, 55)	48 (39, 59.8)	0.43
Cardiac patients	74.7 (24.2)	42.3 (7.8)	0.007*
Balance in the day before SBT (mL)			
Non-cardiac patients	209 (1252)	109 (816)	0.089
Cardiac patients	225 (879)	- 164 (1145)	0.610
Balance in the day of SBT (mL)			
Non-cardiac patients	- 327 (677)	- 136 (806)	0.508
Cardiac patients	- 132 (830)	- 453 (708)	0.74
Central venous pressure (cmH_2_O)			
Non-cardiac patients	8.7 (3.4)	8.9 (3.2)	0.88
Cardiac patients	10.3 (2.1)	8.2(2.4)	0.09
Systolic blood pressure (mmHg)			
Non-cardiac patients	127.3 (16.8)	129.3 (14.5)	0.71
Cardiac patients	122.3 (18.4)	125.6 (19.1)	0.71
Heart rate (beat/minute)			
Non-cardiac patient	94.8 (11.3)	98.9 (21.4)	0.44
Cardiac patients	97.1 (16.5)	90 (23.2)	0.48

*TFC* thoracic fluid content, *SBT* spontaneous breathing trial

*Denotes statistical significance

**Fig. 1:**
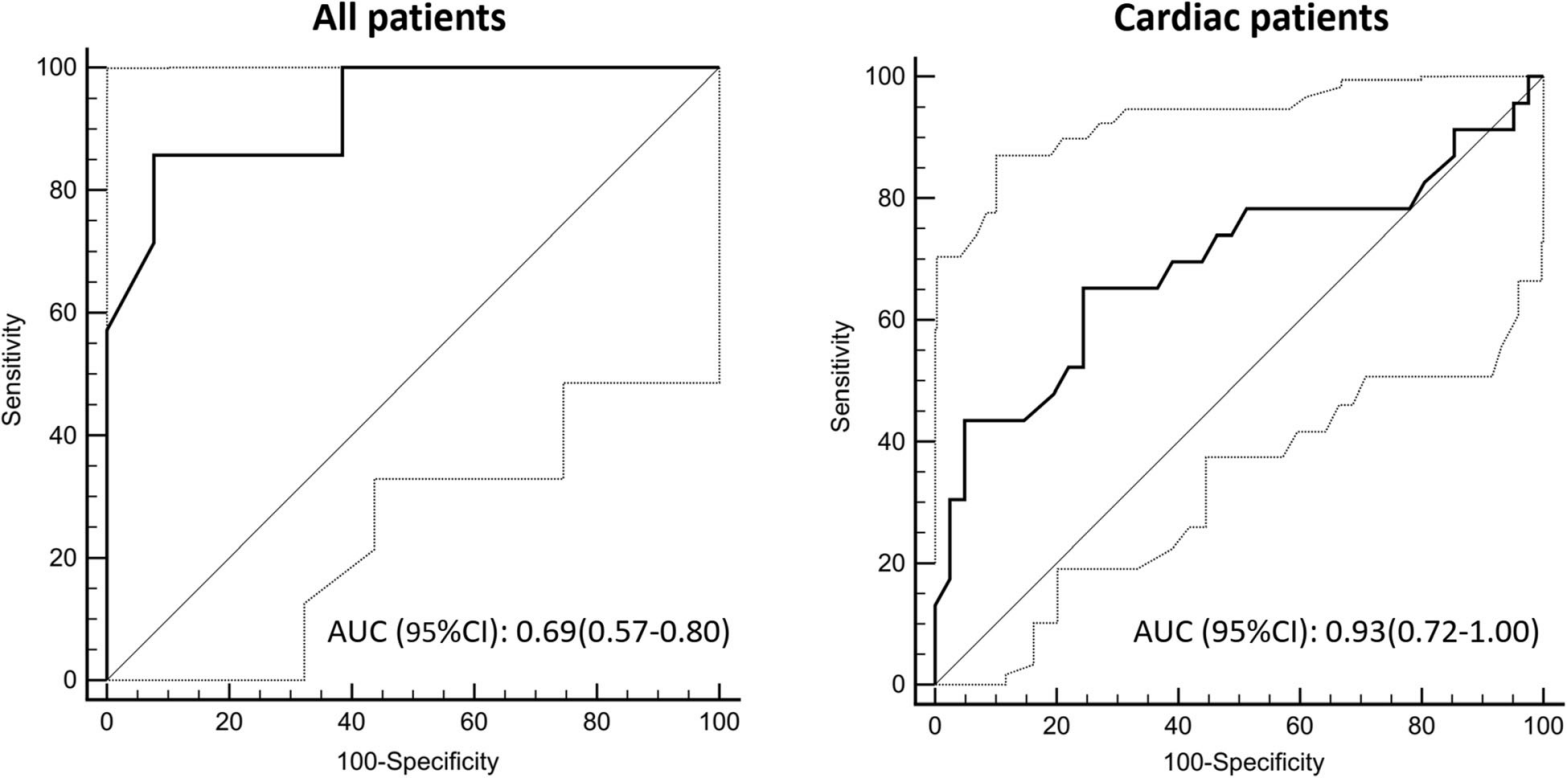
Receiver operating characteristic curve for the ability of thoracic fluid content to predict weaning failure. The solid lines denote the receiver operating curve, and the dashed lines represent the 95% CI. AUC, area under curve; CI, confidence interval

There was a moderate predictive ability for TFC in prediction of weaning failure (AUC [95% CI] 0.69 [0.57–0.8], positive predictive value 60%, negative predictive value 79.5%, cutoff value > 50 kΩ^−1^), while other variables such as P/F ratio, serum HCO3, and RSBI failed to predict weaning failure (Table 4). In the subgroup of cardiac patients, there was an excellent predictive ability for TFC (AUC [95% CI, 0.93 [0.72–1], positive predictive value 85.7%, negative predictive value 92.3%, cutoff value > 50 kΩ^−1^) (Table 5; Fig. 1).

**Table 4:** Accuracy of different variables in predicting weaning failure

Parameter	AUC (95% CI)	Sensitivity	Specificity	PPV	NPV	Cutoff value
TFC	0.69(0.57–0.80)*	65.2%	75.6%	60.0%	79.5%	> 50 kΩ^−1^
Serum HCO3	0.60 (0.47–0.72)	56.5%	68.3%	50.0%	73.7%	> 25 mmol.L^−1^
P/F ratio	0.64 (0.50–0.76)	90.0%	37.5%	41.9%	88.2%	≤ 397.5
RSBI	0.51 (0.38–0.65)	36.8%	72.5%	38.9%	70.7%	> 51.1
Age	0.65 (0.52–0.76)*	82.6%	43.9%	45.2%	81.8%	> 39 years

*AUC* area under curve, *CI* confidence interval, *TFC* thoracic fluid content, *P/F ratio* PaO_2_/fraction of inspired oxygen, *RSBI* rapid shallow breathing index, *PPV* positive predictive value, *NPV* negative predictive value

*Denotes statistical significance

**Table 5:** Accuracy of TFC in predicting weaning failure in cardiac and non-cardiac patients

Parameter	AUC (95% CI)	Sensitivity	Specificity	PPV	NPV	Cutoff value
All patients	0.69 (0.57–0.80)*	65.2%	75.6%	60.0%	79.5%	> 50 kΩ^−1^
Cardiac patients	0.93 (0.72–1.00)*	85.7%	92.3%	85.7%	92.3%	> 50 kΩ^−1^
Non-cardiac patients	0.57 (0.42–0.72)	37.5%	92.9%	75.0%	72.2%	> 65 kΩ^−1^

*AUC* area under curve, *CI* confidence interval, *TFC* thoracic fluid content, *PPV* positive predictive value, *NPV* negative predictive value

*Denotes statistical significance

## Discussion

Thoracic fluid content showed moderate predictive ability for weaning failure; this predictive ability became excellent in the subgroup of patients with impaired systolic function. Measurement of the TFC depends on the impedance cardiography phenomenon which changes according to the resistance of thoracic contents to electric current [9]. The presence of lung congestion is an important cause for weaning failure especially in cardiac patients [12]. Lung congestion may also be triggered by the SBT due to the increase in the left ventricular afterload [13], as well as the increase in the venous return and the subsequent increase in the cardiac preload [14]. The TFC is an index for both extra and intra-vascular thoracic fluid; however, the TFC showed good correlation with ultrasound in estimation of extravascular lung water [10]. Therefore, high TFC value could be an indirect measure of lung congestion and/or hypervolemia which is a known risk factor for failed weaning. This assumption is supported by previous reports in which the TFC was able to follow up the hemodynamic effect of diuretics [15] and to evaluate the thoracic fluid in patients with heart failure [16]. The TFC was also able to follow up the change in the patient body weight and the volume of the ultrafiltrate removed during hemodialysis [17]. The TFC showed good correlation with fluid balance during cardiac surgery [18].

Thoracic fluid content had been classically assessed through thoracic bio-impedance technology. In the last few years, TFC had been also measured using the newer electric cardiometry technology. The electrical cardiometry-based TFC showed useful results in evaluation of the volume status of patients undergoing autologous blood transfusion [9]. In late preterm and term newborn, TFC correlated with the occurrence of respiratory distress in the first 24 h after birth [19]. Our group had recently evaluated TFC in pre-eclamptic mothers and reported that TFC could detect mothers with high risk for pulmonary edema [10]. In the current study, we evaluated TFC in another population, the critically ill patients, and found that the TFC could be a useful tool for detection of weaning failure in cardiac patients.

We reported that the validity of TFC in predicting weaning failure is more significant in patients with impaired systolic function. This is most probably because the impact of lung congestion, represented in our patients by increased TFC, on weaning outcome is usually more prominent in cardiac patients. This explanation is supported by Dessap et al.’s [20] findings in their randomized controlled trial in which they reported that a brain natriuretic peptide-guided protocol for weaning showed better patient outcomes compared to traditional weaning protocol. Dessap et al. had found that the value of brain natriuretic peptide, as a marker of hypervolemia, in guiding weaning is restricted to cardiac patients only [20].

Discontinuation of mechanical ventilation is usually a challenging decision in critical care practice. Premature failed weaning leads to prolonged intubation and increases morbidity and mortality [21]. On the other side, delayed weaning is also associated with serious complications [22]. Daily assessment for readiness-to-wean had been a routine recommendation for every mechanically ventilated patient [23]; however, this approach might be associated with premature extubation leading to weaning failure; thus, meticulous research is continuously conducted for reaching accurate parameters for predicting weaning failure. Various parameters had been previously reported as predictors of lung congestion-related weaning failure such as pulmonary artery occlusion pressure which requires the presence of pulmonary artery catheter [14], impaired left ventricular systolic function [8], and impaired left ventricular diastolic function [24, 25]; both are assessed using echocardiography. Brain natriuretic peptide was reported as another predictor for weaning outcome [7]. In our study, we introduced a simpler parameter that could be measured without the need for blood samples nor operator experience. Thoracic fluid content has many advantages over the previously mentioned predictors for being easily measured by younger physicians, or even by paramedics. Moreover, TFC could be easily interpreted with no inter-observer variability because it is presented in numerical values which have minimal fluctuations. We believe that there is no single parameter that can predict weaning outcome; therefore, assessment of readiness-to-wean should include different factors which evaluate respiratory, cardiovascular, and neurological status of the patient. Thoracic fluid content can help the process of patient weaning especially in cardiac patients who represents 25% of the patients with failed extubation [12]. An elevated TFC could be an alarming sign in cardiac patients to initiate diuretic therapy before extubation.

Our study had some limitations: (1) It is a single center study. (2) All our patients were either trauma patients or emergency surgical patients. Thus, our findings need to be confirmed in future studies in other patient populations. (3) We did not measure cardiac markers such as brain natriuretic peptide and troponin.

## Conclusions

High TFC is associated with failed weaning from mechanical ventilation in critically ill surgical patients. Thoracic fluid content showed moderate ability for predicting weaning outcome in surgical critically ill patients. However, in the subgroup of patients with ejection fraction less than 40%, TFC above 50 kΩ^−1^ showed an excellent ability to predict weaning failure.

## Data Availability

The datasets used and/or analyzed during the current study are available from the corresponding author on reasonable request.

## Abbreviations

AUC: Area under the curve
CI: Confidence interval
ICU: Intensive care unit
P/F ratio: PaO_2_/fraction of inspired oxygen
ROC: Receiver operating characteristic
RSBI: Rapid shallow breathing index
SBT: Spontaneous breathing trial
TFC: Thoracic fluid content
